# Essential competencies for physical therapist managing individuals with spinal muscular atrophy: A delphi study

**DOI:** 10.1371/journal.pone.0249279

**Published:** 2021-04-22

**Authors:** Jean Fitzpatrick Timmerberg, Kristin J. Krosschell, Sally Dunaway Young, David Uher, Chris Yun, Jacqueline Montes

**Affiliations:** 1 Department of Rehabilitation, Columbia University Irving Medical Center, New York City, New York, United States of America; 2 Department of Physical Therapy and Human Movement Sciences and Department of Pediatrics, Northwestern University, Chicago, Illinois, United States of America; 3 Department of Neurology, Division of Neuromuscular Medicine, Stanford University Medical Center, Palo Alto, California, United States of America; 4 Lexa Enterprises, South Kingstown, Rhode Island, United States of America; Kaohsuing Medical University Hospital, TAIWAN

## Abstract

**Background and purpose:**

With the availability and development of disease-modifying therapies for individuals with spinal muscular atrophy (SMA), new emerging phenotypes must be characterized, and potential new treatment paradigms tested. There is an urgent demand to develop an educational program that provides physical therapists (PTs) worldwide the necessary knowledge and training to contribute to best-practice care and clinical research. A competency based education framework is one that would focus on outcomes not process and where progression of learners would occur only after competencies are demonstrated. The first step toward such a framework is defining outcomes. The purpose of this Delphi study was to develop consensus on those competencies deemed essential within the SMA PT community.

**Methods:**

Purposive selection and snowball sampling techniques were used to recruit expert SMA PTs. Three web-based survey rounds were used to achieve consensus, defined as agreement among >80% of respondents. The first round gathered demographic information on participants as well as information on clarity and redundancy on a list of competencies; the second round, collected the same information on the revised list and whether or not participants agreed if the identified domains captured the essence of a SMA PT as well as the definitions for each; and the third asked participants to rank their agreement with each competency.

**Results:**

Consensus revealed 35 competencies, organized under 6 domains, which were deemed essential for a PT working with persons with SMA.

**Discussion:**

In order to develop a curriculum to meet the physical therapy needs of persons with SMA, it is imperative to establish defined outcomes and to achieve consensus on those outcomes within the SMA community.

**Conclusions:**

This study identified essential competencies that will help to provide guidance in development of a formal education program to meet these defined outcomes. This can foster best-practice care and clinical decision-making for all PTs involved in the care of persons with SMA in a clinical and research setting.

## Introduction

Competency-based education (CBE) was first described by McGaghie and colleagues [1] in 1978 but wasn’t until the beginning of the 21^st^ century that it became a widely used approach in post-graduate medical education especially after the introduction of the Canadian Medical Education Directives for Specialist project [2] in the 1990s followed by the Outcome Project of the Accreditation Council for Graduate Medical Education (ACGME) [3] in the United States and the Tomorrow’s Doctor Project in the United Kingdom [4]. Frank et al define CBE as “an approach to preparing physicians for practice that is fundamentally oriented to graduate outcome abilities and organized around competencies derived from an analysis of societal and patient needs. It de-emphasizes time-based training and promises greater accountability, flexibility, and learner-centeredness” [5].

In 2017, The Josiah Macy Foundation, an organization committed to advancing health professions education, held a conference and concluded that CBE can positively impact “our educational and health care systems” [6, 7]. The shift away from the traditional time and process based to an outcome, competency-based educational model in the health professions worldwide, stems from public demand for accountability [8]. The goal of CBE is to provide safer, higher quality care to patients. Progression of learners is completed by the demonstration of competencies and assessments allow coached progression [9]. In this model, time is a resource not a proxy for competence. Competence is assessed, not assumed [9].

While much of the current literature on CBE focuses on medicine it should be applicable to all health professions [7]. The physical therapy profession is currently exploring a paradigm shift toward a CBE model [10, 11]. The National Study of Excellence and Innovation in Physical Therapist Education included recommendations that our profession “Establish a comprehensive, longitudinal approach for standardization of performance-based learning outcomes across the learner continuum that is grounded in foundational domains of professional competence” [12, 13]. Additional sources have identified the need for the profession to develop a framework that defines a set of performance outcomes with common domains that can be utilized across the learner continuum [14, 15]. This continuum would begin upon entrance into a professional education program and end when an individual retires. CBE is a comprehensive approach that begins with looking at the needs of society and continues with determining the competencies that the health care provider must master in order to meet those needs. This facilitates the development of a curriculum which allows the health care provider to achieve mastery of the competencies and a robust program of assessment and mentoring that allows for continued improvement and lifelong learning [7]. Analytic frameworks to define outcomes divide competence into domains. Domains of competence are “broad, distinguishable areas of competence that in the aggregate constitute a general descriptive framework for a profession” [8]. The Accreditation Council for Graduate Medical Education has 8 general competency domains in medical residency education: patient care; knowledge for practice; interprofessional collaboration; interpersonal and communication skills; professionalism; practice-based learning and improvement; systems-based practice, and personal and professional development [8]. Furze et al [14] in a perspective on physical therapy residency and fellowship education recommended 7 domains of competence: Knowledge for practice, communication, clinical reasoning, inquiry skills, systems-based practice, clinical skills and professionalism.

Physical Therapists (PTs) who graduate from professional education programs are prepared for general practice in neurologic and pediatric physical therapy. In 2011, the Academy of Neurologic Physical Therapy identified the neurologic entry-level content for professional education programs integrated with a normative model of PT professional education [16]. The suggested curriculum included the natural course of commonly seen neurologic disorders/diseases. The degenerative disorders listed include Parkinson’s disease, Amyotrophic lateral sclerosis, Huntington disease, and dementias. In 2012, the Academy of Pediatrics held an Education Summit to inform pediatric content in the entry-level PT curriculum. The content included more commonly encountered pediatric conditions including Autism spectrum disorder, cerebral palsy, cystic fibrosis and muscular dystrophy to name a few [17]. Like most practice areas, it is impossible for entry-level physical therapy educational programs to cover all conditions that PTs may encounter when providing care. Spinal Muscular Atrophy (SMA) is often mentioned in pediatric PT courses, but not discussed in great detail.

Spinal Muscular Atrophy is recessively inherited neuromuscular disease affecting lower motor neurons causing muscle weakness and impaired motor function and its severe form, death [18]. The incidence of SMA is 1 in 11,000 births [19]. Because SMA is a rare disorder, it is typically not covered within the entry-level curriculum, therefore PTs working with individuals with SMA would need to have proactively sought continuing education courses and if fortunate, connect with an expert PT for mentorship. Furthermore, with the availability and development of disease-modifying therapies for persons with SMA, there are new emerging phenotypes that must be characterized, and potential new treatment paradigms tested. The majority of PTs are not currently prepared to effectively lead or deliver this effort due to limited clinical expertise and SMA specific educational and research support systems. There is an urgent demand to develop an educational program that would provide PTs worldwide the necessary knowledge and training to contribute to patient care and clinical research.

Defining outcomes (competencies) organized into domains, for PTs working with individuals with SMA is the first step in developing a CBE framework. The purposes of this Delphi study were to develop consensus on (1) a set of competencies (defined outcomes), organized into domains, deemed essential for PTs working with individuals with SMA, and (2) whether the domains of competence and corresponding definitions represent the essence of a PT.

## Materials and methods

Columbia University IRB approved this study- Protocol # AAAS7994. By completing the survey, participants indicated they were providing informed consent. All participants were provided an informational sheet prior to the onset of the study.

An initial meeting was held in Copenhagen over a two day period with 13 international PT SMA experts*, who were currently practicing as clinicians treating individuals with SMA and/or engaged as evaluators in SMA clinical research trials. The experts developed a list of 66 competencies organized under 7 broad areas or domains of competence: Knowledge, Assessment, Clinical Reasoning, Communication, Professionalism, Management, and Leadership. The Delphi method, which engages a group of participants or experts over multiple rounds of surveys to establish consensus on a particular topic of interest was then utilized to obtain consensus on the list created [20–26]. The list of competencies was presented to a larger group of SMA expert participants. Both purposive selection and snowball sampling techniques were used to recruit the larger group of PTs who met the inclusion criteria that they were a licensed physical therapist in their country of residence and that they were involved in the management / evaluation of individuals with SMA. The survey rounds were conducted in an online format through RedCap, a secure web application for building and managing online surveys and databases (Vanderbilt University, Nashville, TN, USA).

### Round one

The first-round survey (S1 Appendix) was developed by the research team and reviewed by 2 individuals with experience in qualitative educational research. Feedback and assistance were provided to finalize the survey and instructions. Demographic information was collected on all participants including their geographic location, primary areas of work, and the ages and phenotypes of the patients with SMA typically seen. The complete list of competencies was provided and for each competency, participants were asked if the item was clear or redundant. If an item was deemed unclear, participants were asked to provide feedback. Participants identified redundant items. Participants were also asked if they felt competencies were missing.

Feedback was independently reviewed by three SMA experts (KK, SDY, JM) on the research team and coded for areas of commonality and consistency. The suggested modifications were then brought to the research team. Any areas of difference were discussed and agreed upon. When multiple participants (>2) identified redundancies in the competencies listed, that particular competency was removed.

### Round two

In the second round, participants were provided the revised list of competencies within 6 domains along with a description for each domain. Participants were once again asked to provide feedback as to whether each competency was clear and if it was redundant with another competency. Participants were also asked if the identified domains captured the essence of a SMA physical therapist, whether or not they agreed with the definitions provided for each and if competencies were listed under the appropriate domain (S2 Appendix).

Feedback from round 2 was again independently reviewed by the three SMA experts on the research team who then brought the suggested modifications to the larger group discussion. Any areas of difference were discussed and agreed upon. Modifications centered on clarification and consistency of language throughout with the revised list.

### Round three

In the third and final round, participants were asked to rank their agreement with each competency provided in the final list as being essential using a 5-point Likert scale. Consensus was defined a priori as ≥ 80% of respondents rating a competency as Agree or Strongly Agree as essential for a PT to demonstrate mastery in before working with individuals with SMA. Participants also had the opportunity to provide comments about any of the competencies listed under a given domain as well as any additional comments or suggestions. In addition to the audit trail, trustworthiness was supported by allowing participants to review and revise the data during the study. The achievement of consensus for items in the final rounds also served as a check on the data (S3 Appendix).

### Data analysis

Descriptive statistics, including frequencies, percentages, and ranges, were used to analyze demographic data. Data collected from responses related to clarity and redundancy in Rounds 1 and 2 were analyzed using methods typical of qualitative data analysis [20–26]; an example of such is provided in Table 1. This qualitative data analysis included three members of the research team reading all of the feedback independently, identification of themes or patterns to the feedback and organization of that feedback into categories: for example feedback related to clarity, redundancy, or missing items. Data from round 3 were analyzed using the percentages of responses for each category on the 5-point Likert-scale.

**Table 1:** Characteristics of participants.

Geographic Location	N
Inside US	72
Outside US	28
**Professional Area**	
Academia ONLY (Teaching)	13
Academic (Research)	31
Industry Sponsored (Teaching Training)	22
Clinical Trial Evaluation	48
Other Clinical Research Evaluations	50
Multidisciplinary Evaluation / Management Clinic	71
Treatment Based Clinic	65
**Ages Treated**	
Infants	76
Toddlers	86
Children	86
Adolescents	86
Young Adults	47
Adults	21
**Phenotypes Treated**	
Pre-symptomatic	40
Non-sitters	91
Sitters	95
Walkers	83

## Results

Demographics: Respondents (N = 100) represented a group of expert PTs working with individuals with SMA from across the globe (Table 1). Fifteen countries along with 26 states from the US were represented in this study. They were primarily PTs who worked inside the US in either a multidisciplinary evaluation / management or treatment-based clinic. The majority worked with patients under the age of 18 with various phenotypes.

### Round one

Surveys were distributed to 321 potential participants. The response rate was 31.2%, with 100 Round 1 surveys returned. Table 2 represents the distribution of respondents who completed each round’s survey. Responses were reviewed independently by three members of the research team who are also experienced clinicians working with individuals with SMA. When multiple participants (>2) identified redundancies in the competencies listed, researchers reviewed the information and consolidated similar statements to reduce redundancy. Additionally, items requiring clarity were reworded to eliminate confusion. Competencies were not lost or deemed unimportant, but were better captured in or consolidated within another competency. This process collapsed the list from 66 to 35 competencies and from 7 to 6 domains. The six remaining domains were knowledge for practice (KP), patient management (PM), communication (C), critical reasoning (CR), professionalism (P), and education (E). Table 3 provides an example of an audit trail of the analysis used in Round 1.

**Table 2:** Response rate by round.

Round	Number Provided the Survey	Respondents	Response Rate
1	321	100	31.2%
2	100	77	77%
3	77	62	80.5%

**Table 3:** Example audit trail of analysis for knowledge of practice domain.

Competencies Provided in Round 1	Edits Made Based on Feedback	Revised Competency List for Round 2
1. Demonstrates an understanding of the pathophysiology of SMA.		1. Demonstrates an understanding of the pathophysiology of SMA.
2. Demonstrates an understanding of the natural history of SMA.	Deleted “natural history,” added “disease progression over time and its impact on body functions and structure, including all body systems (ie musculoskeletal, respiratory, cardiovascular, digestive, neurological)”	2. Demonstrates an understanding of the SMA disease progression over time and its impact on body functions and structure, including all body systems (e.g., musculoskeletal, respiratory, cardiovascular, digestive, neurological).
3. Demonstrates an understanding of standards of care.	Deleted “standards of care,” added “the multidisciplinary care guidelines for SMA and how they can be applied within one’s (provider/patient’s) current healthcare system.”	3. Demonstrates an understanding of the multidisciplinary care guidelines for SMA and how they can be applied within one’s (provider/patient’s) current healthcare system.
4. Demonstrates an understanding of local application of standards of care.	Combined in 3	
5. Demonstrates an understanding of pharmacologic treatments and their mechanism of action.	Reworded to: Demonstrates an understanding of the mechanism of action (pharmacodynamics) of pharmacologic treatments	4. Demonstrates an understanding of the mechanism of action (pharmacodynamics) of pharmacologic treatments.
6. Demonstrates an understanding of rehabilitation treatment modalities.	Reworded to: Demonstrates an understanding of physiotherapy care guidelines with regards to signs/symptoms, evaluations, and interventions	5. Demonstrates an understanding of physiotherapy care guidelines with regard to signs/symptoms, evaluations, and interventions.
7. Demonstrates an understanding of impact on body systems in SMA.	Merged into #2	
8. Demonstrates an understanding of impact of comorbidities on the individual with SMA.	Merged into #2	
9. Demonstrates an understanding of assessments of impairments.	Removed 9–11 as it is now within #6	
10. Demonstrates an understanding of impact of age/stage/phenotype/resources on the management of SMA.	Now within #6	
11. Demonstrates an understanding of available orthotics, bracing, mobility devices, positioning aids, equipment and environmental modifications for ADLs.	Now within #6	
12. Demonstrates an understanding of typical development and aging across lifespan.	Reworded to: Demonstrates an understanding of typical development of the healthy individual and aging across lifespan	6. Demonstrates an understanding of typical development of the healthy individual and aging across the lifespan.
13. Demonstrates an understanding of medical management including imaging, diagnostics, labs, etc.	Now within #3	
14. Demonstrates an understanding of clinical presentations of SMA (signs and symptoms).	Now contained within #6	
15. Is able to recognize evolving phenotypes.	Reworded to: Recognizes evolving phenotypes as classic SMA trajectories diverge from expected disease progression as a result of pharmacologic or other interventions	7. Recognizes evolving phenotypes as classic SMA trajectories diverge from expected disease progression as a result of pharmacologic or other interventions.

### Round two

A revised list of 35 competencies under 6 domains were returned to the 100 participants, who participated in Round 1. In this second round, 77 surveys (77%) were completed and returned. Participants commented on competencies needing clarification or considered redundant. Responses were reviewed and clarifications and eliminations were executed by consensus of the research team. One hundred percent of participants felt the six domains captured the essence of an SMA PT. When discussing the domain definitions, 87.0% were in agreement for the definition of KP, 90.9% for PM, 96.1% for C, CR, and P, and 93.5% for E.

### Round three

In Round 3, participants who completed Round 2 were sent a survey and indicated their level of agreement on a 5-point Likert scale, ranging from Strongly Disagree to Strongly Agree, regarding each competency as important or essential for an individual PT working with individuals with SMA. Seventy-seven surveys were distributed and 62 (80.5%) were returned. All 35 competencies achieved >80% agreement that they were essential competencies of a PT working with an individual with SMA. The final list of competencies organized within the 6 domains, including the title and description, can be found in Table 4.

**Table 4:** Essential competencies for physical therapists working with individuals with spinal muscular atrophy organized within six domains.

Domain of Competence	Domain Description	Competencies
Knowledge of Practice	*Physiotherapists are experts in movement and function*. *They demonstrate knowledge in the established and evolving evidence-based science related to SMA*. *Physiotherapists integrate this unique knowledge and skills to provide quality care and enhance the participation*, *health and wellbeing of their patients with SMA*.^*1*^	1. Demonstrates an understanding of typical development of the healthy individual and aging across the lifespan. 2. Demonstrates an understanding of the pathophysiology of SMA. (i.e., SMN function within the cell, neuromuscular junction pathophysiologic abnormalities in SMA, mitochondrial function in SMA, spinal cord pathology in SMA, muscle pathology in SMA) 3. Demonstrates an understanding of the SMA disease progression over time in each individual, and its impact on body functions and structure, including all body systems (e.g., musculoskeletal, respiratory, cardiovascular, gastrointestinal, neurological). 4. Demonstrates an understanding of the multidisciplinary care guidelines for SMA and how they can be applied within one’s (provider/patient’s) current healthcare system. 5. Demonstrates an understanding of physiotherapy care guidelines for SMA with regard to signs/symptoms, evaluations, and interventions. 6. Demonstrates an understanding of the mechanism of action (pharmacodynamics) of pharmacologic treatments for SMA. 7. Recognizes evolving phenotypes as newly-observed patterns of SMA disease progression diverge from classic SMA trajectories as a result of pharmacologic or other interventions.
Patient Management	*As experts in movement and function*, *physiotherapists provide care for individuals with SMA through the use of knowledge*, *skills and shared decision making with patients and families and other professionals to optimize patients’ outcomes*.^*2*,*3*^	8. Demonstrates active listening skills throughout all aspects of SMA patient management. 9. Gathers a comprehensive medical and psychosocial history pertinent to SMA from the patient/family. 10. Selects and administers appropriate, comprehensive impairment-based, functional, and participation assessments for individuals with SMA in a standardized, safe and reliable manner. 11. Interprets and applies results of the impairment-based, functional, and participation assessments to the management of the individual with SMA. 12. Recommends appropriate assistive devices, seating and mobility equipment, and environmental modifications for the individual with SMA to optimize function. 13. Provides patient/family-centered management. 14. Makes appropriate referrals to members of the multi-disciplinary team. 15. Applies physiotherapy care guidelines for SMA patient management across the disease spectrum and lifespan. 16. Demonstrates safe handling skills during physiotherapy management of the individual with SMA across the disease spectrum and lifespan.
Communication	*As strong communicators*, *physiotherapists providing care for individuals with SMA demonstrate interpersonal*, *verbal*, *nonverbal and written communication skills to effectively exchange information and collaborate with patients*, *families*, *and other professionals*.^*4*^	17. Clearly and accurately receives and disseminates information in a respectful manner that considers situational needs. 18. Effectively engages in interprofessional communication that positively affects patient outcomes. 19. Adapts to diverse verbal and non-verbal communication styles during anticipated and unanticipated patient and professional interactions. 20. Selects and incorporates appropriate strategies to manage challenging encounters with patients and others.
Clinical Reasoning	*Physiotherapists can critically translate evidence-based knowledge into practice*. *They demonstrate the ability to organize*, *synthesize*, *integrate*, *and apply sound clinical rationale for SMA patient management*^*5*^	21. Synthesizes information gathered during the examination to form a movement diagnosis related to SMA. 22. Utilizes current best practice guidelines for management of patients with SMA to interpret examination findings and to develop shared goals and treatment priorities with patient/family input. 23. Incorporates SMA evidence-based practice to determine treatment procedures and progression of intervention. 24. Anticipates future scenarios with individuals with SMA based on an understanding of disease progression and impact of pharmaceutical and rehabilitation treatments. 25. Considers factors that may influence decision making with regards to pharmaceutical and rehabilitation treatments for SMA, and responds with a patient/family-centered focus.
Professionalism	*Physiotherapists providing care for individuals with SMA*, *demonstrate a commitment to life-long learning while working in the best interest of patients*, *colleagues*, *society and the profession*. *They maintain high standards of behavior*, *exhibit appropriate professional conduct*, *advocate for the patient*, *and adhere to ethical principles*.^*2*,*4*^	26. Identifies resources and pursues areas of professional development that lead to continued competence in SMA. 27. Effectively and regularly utilizes external feedback and self-reflection to improve SMA patient care. 28. Consistently demonstrates values of diversity, equality and inclusion in interactions with individuals with SMA and their families. 29. Understands the potential impact of ethical issues in SMA on patient outcomes, patient/therapist safety and public trust and works to develop and implement solutions. 30. Promotes innovation in SMA research and practice to advance the profession. 31. Serves as a SMA resource to provide feedback and expert guidance to the interprofessional community.
Education	*All physiotherapists are educators*, *teaching and mentoring various members of the SMA community including patients*, *families*, *students*, *and other professionals*.	32. Designs and directs educational activities for various members of the SMA community (including patients, families, students and other professionals) 33. Implements effective teaching strategies for the various domains of learning (cognitive- knowledge, psychomotor- hands on skills, and affective-moods, feelings, and attitudes). 34. Adapts one’s teaching style to reflect the learner: their level of experience, preferences, needs and goals. 35. Provides mentorship to advance the professional development of other SMA physiotherapists.

## Discussion

With the availability and development of disease-modifying therapies for individuals with SMA, there are new emerging phenotypes that must be characterized, and new treatment paradigms tested. Moreover, given the rapidly growing field of SMA treatments, there is an urgent need to ensure the prescribed standards of care are delivered to all individuals with SMA regardless of age, clinical trial participation, treatment, or region. However, the majority of PTs are not currently prepared to deliver this service at this time because they lack the necessary knowledge, skills and ability for mentored practice. In part, this deficiency may be addressed through appropriate education resources that are accessible to all and can deliver precise details on standards for both assessment and management. There is an urgent demand to develop an educational program that would provide PTs worldwide the necessary knowledge and skills while allowing adequate practice to allow a learner to be able to independently provide safe and effective patient care as well as contribute to related clinical research.

Competency-based education is an approach that is based on the community and patient needs that has been adopted in medicine and other health professions including physical therapy. The SMA Teaching and Excellence for Physiotherapists; International Network (STEP IN: https://www.stepinsma.org/) initiative is a program that intends to utilize the results of this study. Employing the competencies that were deemed essential in this Delphi study, STEP IN intends to build a curriculum that will allow learners to demonstrate these competencies. A needs assessment highlighted the demand for education and practical guidance on the physical therapy assessment and management of individuals with SMA. STEP IN aims to address the current unmet need for expertise and resources to provide outstanding care for individuals with SMA across the lifespan and phenotypic spectrum around the world through a comprehensive competency-based education program. The “outcomes” of such a program will be based on the community and patient needs that have been adopted in medicine and other health professions including physical therapy. This is a novel solution that, once developed and implemented in SMA, could also serve as a template or be integrated into a broader program for other neuromuscular disorders (NMD).

A CBE framework reassures the community that PTs are capable of safely providing high quality care to individuals with SMA and NMD. NMD is a group of rare disorders seldom covered in detail in entry-level PT education. Existing educational opportunities (1) are not based on consensus defined outcomes and (2) do not ensure that participants have learned and are able to apply the knowledge in the workplace. With the availability of disease modifying therapies, there is a growing demand on non-specialist PTs to manage and treat these individuals. Therefore, post-graduate NMD specific PT training is paramount.

This study used the Delphi method to attain consensus among SMA PTs on essential competencies that all PTs should master prior to working with individuals with SMA regardless of country where practicing or years of experience as a PT. The Delphi method was a successful design for this study, allowing experts in the SMA community to contribute to consensus development [21]. While there was a larger representation of PTs from inside the US (72%) than the percentage of PTs outside the US (28%), nearly one third of the participants came from outside of the US and represented 2 continents (Europe and Australia) and 14 countries. Greater representation from PTs in the US was in part due to the need for English-speaking participants. Future work will include translated materials to permit participation of PTs from countries not represented in this study including those in Eastern Europe, Asia, and South America.

The final 35 competencies organized into 6 domains reached a minimum of 80% consensus among the participants as essential for all PTs to master prior to working with individuals with SMA. The domains of competence were deemed representative of the essence of the PT profession. When a learner has multiple competencies under one domain where they are challenged in achieving mastery, faculty can utilize this information to provide specific feedback to learners, therefore customizing remediation to maximize potential success. Four of the six domains named in this study align well with the domains established by the Association of American Medical Colleges for the progression to medical residency [27], and 5 of the six domains aligned within six of the domains proposed by Furze et al. [14]. The competencies and domains listed by AAMC are for students entering residency. They therefore would continue to have supervision and guidance. The 35 competencies that achieved consensus in this study, organized under the 6 domains, are for licensed PTs who are autonomous practitioners and should be of a higher or advanced level.

## Conclusions

The results of this study provide the initial framework for developing a curriculum specifically targeted for SMA PTs. Future work should focus on the development of the curriculum and educational program. CBE is an approach that is outcome based instead of process-based, where competence is assessed not assumed. An implementation plan for an international SMA competency-based education program and a path to extrapolate this program to other neuromuscular conditions is needed.

## Supporting information

S1 Appendix Round one survey. (DOCX)

S2 Appendix Round two survey. (DOCX)

S3 Appendix Round three survey. (DOCX)
