# Supplementary material for: Essential competencies for physical therapist managing individuals with spinal muscular atrophy: A delphi study
Source: PLoS One. 2021 Apr 22;16(4):e0249279. doi: 10.1371/journal.pone.0249279 (PMC8062020; doi:10.1371/journal.pone.0249279)
Supplement: S1 Appendix — (DOCX) [file pone.0249279.s001.docx]

Survey Round 1

With the availability and development of disease-modifying therapies for individuals with spinal muscular atrophy (SMA), there are new emerging phenotypes that must be characterized, and potential new treatment paradigms tested. Majority of physiotherapists (PTs) are not currently prepared to lead this effort. There is an urgent demand to develop an educational program that would provide PTs worldwide the necessary knowledge and training to contribute to patient care and clinical research. Before such a program can be developed it is necessary to define the outcomes that the learners will need to show competence in. Expert SMA PTs from five countries met in Copenhagen and developed a list of competencies they believe are essential for an individual working with individuals with SMA to demonstrate competence in. The competencies were categorized into seven themes: knowledge, assessment, clinical reasoning, communication, professionalism, management and leadership. While the development of this list was an crucial first step, it is important to come to consensus on the list among SMA experts worldwide.

You have been selected for participation in a modified Delphi survey secondary to your experience and expertise in the clinical management and or evaluation of individuals with Spinal Muscular Atrophy (SMA). This study involves the use of an on-line survey via RedCap where subject data will be stored by code. Only the Primary Investigator will have access to the list linking the code to a participant. Study results however will be reported in aggregate, not by individual responses. Therefore, no data will be reported that can be linked to you. While there are no direct benefits to your participation in this study, the information is crucial in supporting the education of physical therapists working with individuals with SMA.

The modified Delphi study will consist of a series of on-line surveys, that will occur over approximately 3 months, asking specific questions related to the draft list of essential competencies. In the first round you will be asked to evaluate each competency and indicate whether it is clearly written, if it is redundant with another competency and if there are any competency items missing. The list will be modified based on overall feedback. In subsequent rounds, the list will be returned to you to indicate whether or not you agree that each item is essential for a physiotherapist to demonstrate competence prior to working with individuals with SMA.

If you agree to participate in this study please complete this on line survey. By agreeing to participate, you are indicating that you are giving informed consent. Your participation is completely voluntary, you can choose to decline at any point, even right before the study is set to begin. Therefore participation status will NOT have any effect on future collaborations.

If you have questions at any time feel free to contact the primary investigators:

Jacqueline Montes, PT, EdD

Associate Professor of Rehabilitation and Regenerative Medicine in the Programs in Physical Therapy

Columbia University Irving Medical Center

617 West 168th Street, Room 347

New York, NY 10032

212-305-8916 phone

[jm598@cumc.columbia.edu](mailto:jm598@cumc.columbia.edu)

Jean Fitzpatrick Timmerberg, PT, PhD, MHS

Associate Director Programs in Physical Therapy

Vagelos College of Physicians & Surgeons

617 West 168th Street, Georgian Building - 3rd Floor

New York, NY 10032

(212) 305-2814 phone

[jt2634@cumc.columbia.edu](mailto:jt2634@cumc.columbia.edu)

Demographic Questions:

1- Please indicate your primary geographic location:

a) Country

If outside the US: CITY

If within the US: STATE

2- Please check ALL the areas that you consistently have been involved in over the past 2 years:

a) Academic ONLY (teaching)

b) Academic – Research

c) Industry Sponsored – Teaching Training

d) Clinical Trial Evaluations (industry)

e) Other Clinical Research Evaluations

f) Multidisciplinary Evaluation / Management Clinic

g) Treatment Based Clinic

3- Please indicate the ages of the patients with SMA that you typically work with:

(CHECK ALL THAT APPLY)

1. Infants (<2 years)
2. Toddlers (2-4 years)
3. Children (>4 – 10 years)
4. Adolescents (>10 – 18 years)
5. Young Adults (>18 – 40 years)
6. Adults (>40 years)

4- Please indicate the phenotype of the patients with SMA that you typically work with: (CHECK ALL THAT APPLY)

1. Pre-symptomatic
2. Non-sitters
3. Sitters
4. Walkers

For each of the competencies listed, you will be asked to answer the following questions:

Are the competencies below clear (yes / no)? If no – why?

Are the competencies below redundant? (yes / no). If no – which item?

List of Competencies

**Knowledge**

1. Demonstrates an understanding of the pathophysiology of SMA
2. Demonstrates an understanding of the natural history of SMA
3. Demonstrates an understanding of standards of care
4. Demonstrates an understanding of local application of standards of care
5. Demonstrates an understanding of pharmacologic treatments and their mechanism of action
6. Demonstrates an understanding of rehabilitation treatment modalities
7. Demonstrates an understanding of impact on body systems in SMA
8. Demonstrates an understanding of impact of comorbidities on the individual with
9. Demonstrates an understanding of assessments of impairments
10. Demonstrates an understanding of impact of age/stage/phenotype/resources on the management of SMA
11. Demonstrates an understanding of available orthotics, bracing, mobility devices, positioning aids, equipment and environmental modifications for ADLs
12. Demonstrates an understanding of typical development and aging across lifespan
13. Demonstrates an understanding of medical management including imaging, diagnostics, labs, etc.
14. Demonstrates an understanding of clinical presentations of SMA (signs and symptoms)
15. Is able to recognize evolving phenotypes

**Assessment**

1. Is able to administer a comprehensive subjective exam
2. Is able to administer a comprehensive functional assessment
3. Is able to administer a comprehensive impairment assessment
4. Is able to administer functional assessments in a safe and standardised fashion
5. Is able to administer impairment assessments in a safe and standardised fashion
6. Is able to assess the need for and the use of appropriate orthotics, bracing, mobility devices, positioning aids, equipment and environmental modifications for ADLs

**Clinical Reasoning**

1. Is able to identify appropriate treatment goals
2. Is able to assess and modify treatment programs based on individual presentation
3. Is able to interpret assessment findings
4. Is able to identify the appropriate orthotics, bracing, mobility devices, positioning aids, equipment and environmental modifications for ADLs
5. Is able to identify the need for intra/inter-professional referrals within and outside their facility
6. Is able to recognize the need for evidence-based practice to support patient care

**Communication**

1. Is able to adapt communication style depending on audience and setting
2. Is able to communicate clearly and effectively
3. Is able to demonstrate appropriate verbal communication in all interactions with individuals and families
4. Is able to demonstrate appropriate non-verbal communication in all interactions with individuals and families
5. Is able to demonstrate appropriate written communication in all interactions with individuals and families
6. Is able to demonstrate appropriate verbal communication in all interactions with HCPs and non-HCPs involved in the patient care
7. Is able to demonstrate appropriate non-verbal communication in all interactions with HCPs and non-HCPs involved in the patient care
8. Is able to demonstrate appropriate written communication in all interactions with HCPs and non-HCPs involved in the patient care
9. Is able to demonstrate active listening skills
10. Is able to use communication tools and technologies effectively
11. Is able to communicate with empathy and respect
12. Is able to provide constructive feedback to various SMA care providers

**Professionalism**

1. Is able to recognize and practice individual-centered care
2. Is able to commit to lifelong learning
3. Is able to participate in SMA community service at a local, regional and/or international level
4. Is able to demonstrate respect for culture/belief systems
5. Is able to behave with honesty and respect for others
6. Is able to behave in a manner that values diversity
7. Is able to adhere to the appropriate code of ethics
8. Is able to integrate self-reflection and external feedback to improve individual practice

**Management**

1. Is able to apply individual-centered care
2. Is able to make intra/inter-professional referrals within and outside their facility
3. Is able to apply evidence-based practice to patient care
4. Is able to manage individual and family expectations
5. Is able to manage individuals with a non-complex presentation in the following groups; infant, child, adolescent, adult
6. Is able to manage individuals with a complex presentation in the following groups: infant, child, adolescent, adult
7. Is able to demonstrate safe handling skills
8. Is able to manage individuals based on functional status
9. Is able to disseminate appropriate information to all members of the SMA community

**Leadership**

1. Is able to contribute to the learning of various members of the SMA community
2. Is able to create opportunities for professional development
3. Is able to contribute to the advancement of the profession
4. Is able to promote innovation in healthcare
5. Is able to promote innovation in clinical research
6. Is able to assess learners of all backgrounds
7. Is able to contribute to a publication submission
8. Is able to contribute to the preparation of a grant submission
9. Is able to present at a local, national and/or international conference
10. Is able to serve on an advisory board

Is there anything missing? Please comment below:
