# Supplementary material for: Essential competencies for physical therapist managing individuals with spinal muscular atrophy: A delphi study
Source: PLoS One. 2021 Apr 22;16(4):e0249279. doi: 10.1371/journal.pone.0249279 (PMC8062020; doi:10.1371/journal.pone.0249279)
Supplement: S2 Appendix — (DOCX) [file pone.0249279.s002.docx]

Survey- Round Two

Thank you for your feedback and comments in the first round. All of the feedback was reviewed by the research team and the list was modified accordingly. There are now 35 competencies under 6 domains.

In this second round we ask for your input on two areas: the domains of competence listed and their definitions, and feedback on clarity and redundancy on the updated list of competencies. We ask that you complete this survey by Tuesday, May 26th, 2020. We truly appreciate your time and expertise!

If you have questions at any time feel free to contact the primary investigators:

Jacqueline Montes, PT, EdD

Associate Professor of Rehabilitation and Regenerative Medicine in the Programs in Physical Therapy

Columbia University Irving Medical Center

617 West 168th Street, Room 347

New York, NY 10032

212-305-8916 phone

[Jm598@cumc.columbia.edu](mailto:Jm598@cumc.columbia.edu)

Jean Fitzpatrick Timmerberg, PT, PhD, MHS

Associate Director

Programs in Physical Therapy, Vagelos College of Physicians & Surgeons

617 West 168th Street, Georgian Building - 3rd Floor

New York, NY 10032

212-305-2814 phone

[jt2634@cumc.columbia.edu](mailto:jt2634@cumc.columbia.edu)

If you would like to download a PDF version of the domains and competencies, you can do so here:

Domains of competence are broad, distinguishable areas that in the aggregate constitute a general descriptive framework for a profession- in particular, SMA physiotherapists. It's helpful to know the areas in which we want to be able to assess the abilities of individuals but ultimately the core of what we are assessing are the competencies within those domains.

The competencies within this study have been organized into 6 domains named and defined as follows:

1) Knowledge of Practice (KP): As experts in mobility and function, physiotherapists demonstrate knowledge in the established and evolving science related to SMA and integrate the unique knowledge, skills and attitudes to provide quality care and enhance the health and wellbeing of their patients with SMA.^1^

2) Patient Management (PM): As experts in movement and function, physiotherapists provide care for individuals with SMA through the use of foundational skills and shared decision making with patients and families to optimize patients' outcomes.^2, 3^

3) Communication (C): As strong communicators, physiotherapists providing care for individuals with SMA demonstrate interpersonal and verbal, nonverbal and written communication skills to effectively exchange information and collaborate with patients, their families, and health professionals.^4^

4) Clinical Reasoning (CR): Physiotherapists demonstrate the ability to organize, synthesize, integrate, and apply sound clinical rationale for SMA patient management.^5^

5) Professionalism (P): Physiotherapists providing care for individuals with SMA, demonstrate a commitment to working in the best interest of patients, colleagues, society and the profession by maintaining high standards of behavior, exhibiting appropriate professional conduct, advocating for the patient, and adhering to ethical principles.^2, 4^

6) Education (E): All physiotherapists are educators, teaching and mentoring students and various members of the SMA community including patients, families, other clinicians, and researchers.

Questions:

• Do you feel that the six domains capture the essence of a SMA physiotherapist? YES/NO, Leave a comment

• Do you agree with the definition for Knowledge of Practice (KP)? YES/NO, Leave a comment

• Do you agree with the definition for Patient Management (PM)? YES/NO, Leave a comment

• Do you agree with the definition for Communication (C)? YES/NO, Leave a comment

• Do you agree with the definition for Clinical Reasoning (CR)? YES/NO, Leave a comment

• Do you agree with the definition for Professionalism (P)? YES/NO, Leave a comment

• Do you agree with the definition for Education (E)? YES/NO, Leave a comment

1. National Physiotherapy Advisory Group. Competency Profile for Physiotherapists in Canada (2017). https://www.peacaepc.ca/pdfs/Resources/Competency%20Profiles/Competency%20Profile% 20for%

20PTs%202017%20EN.pdf retrieved 3/30/20

2. Education Leadership Partnership. Essential Competencies for Entrance into Practice. Accessed 4/27/20.

3. Grignon TP, Henley E, Lee KM, Abentroth MJ, Jette DU. Expected Graduate Outcomes in US Physical Therapist

Education Prorams: A Qualitative Study.JPTE. 2014;28(1) 48-57

4. Englander R, Cameron T, Ballard A J, Dodge J, Bull J, Aschenbrener AC. Toward a Common Taxonomy of

Competency Domans for Health Professions and Competencies for Physicians. Acad Med. 2013;88(8) 1088- 1094.

5. American Physical Therapy Association. Core Competencies of a Physical Therapist Resident. Accessed 4/4/20

1. Do you feel that the six domains capture the essence of a SMA physiotherapist?

Yes /No

Comments:

1. Knowledge of Practice Domain

Knowledge of Practice (KP): As experts in mobility and function, physiotherapists demonstrate knowledge in the established and evolving science related to SMA and integrate the unique knowledge, skills and attitudes to provide quality care and enhance the health and wellbeing of their patients with SMA.^1^

Do you agree with the definition for Knowledge of Practice (KP)?

Yes /No

Comments:

1. Patient Management Domain

Patient Management (PM): As experts in movement and function, physiotherapists provide care for individuals with SMA through the use of foundational skills and shared decision making with patients and families to optimize

patients' outcomes.^2, 3^

Do you agree with the definition for Patient Management (PM)?

Yes /No

Comments:

1. Communication Domain

Communication (C): As strong communicators, physiotherapists providing care for individuals with SMA demonstrate interpersonal and verbal, nonverbal and written communication skills to effectively exchange information and collaborate with patients, their families, and health professionals.^4^

Do you agree with the definition for Communication (C)?

Yes /No

Comments:

1. Clinical Reasoning Domain

Clinical Reasoning (CR): Physiotherapists demonstrate the ability to organize, synthesize, integrate, and apply sound clinical rationale for SMA patient management.^5^

Do you agree with the definition for Clinical Reasoning (CR)?

Yes /No

Comments:

1. Professionalism Domain

Professionalism (P): Physiotherapists providing care for individuals with SMA, demonstrate a commitment to working in the best interest of patients, colleagues, society and the profession by maintaining high standards of behavior, exhibiting appropriate professional conduct, advocating for the patient, and adhering to ethical principles.^2, 4^

Do you agree with the definition for Professionalism (P)?

Yes /No

Comments:

1. Education Domain

Education (E): All physiotherapists are educators, teaching and mentoring students and various members of the SMA community including patients, families, other clinicians, and researchers.

Do you agree with the definition for Education (E)?

Yes /No

Comments:

Thank you for your feedback and comments related to clarity and redundancy in the first round. We also appreciated your additional comments on missing competencies. All of the feedback was reviewed by the research team and the list was modified accordingly. There are now 35 competencies under the 6 domains.

For each competency, please indicate whether it is written clearly, is redundant with another and if you believe it is listed under the appropriate domain of competence.

**Knowledge of Practice (KP) Competencies**

1. Demonstrates an understanding of typical development of the healthy individual and aging across the lifespan.
2. Demonstrates an understanding of the pathophysiology of SMA.
3. Demonstrates an understanding of the SMA disease progression over time and its impact on body functions and structure, including all body systems (e.g., musculoskeletal, respiratory, cardiovascular, digestive, neurological).
4. Demonstrates an understanding of the multidisciplinary care guidelines for SMA and how they can be applied within one's (provider/patient's) current healthcare system.
5. Demonstrates an understanding of physiotherapy care guidelines with regard to signs/symptoms, evaluations, and interventions.
6. Demonstrates an understanding of the mechanism of action (pharmacodynamics) of pharmacologic treatments.
7. Recognizes evolving phenotypes as classic SMA trajectories diverge from expected disease progression as a result of pharmacologic or other interventions.

Do you have any general comments about the competencies in the Knowledge section?

**Patient Management Competencies**

1. Demonstrates active listening skills throughout all aspects of SMA patient management.
2. Gathers a comprehensive medical and psychosocial history pertinent to SMA from the patient/family.
3. Selects and administers comprehensive impairment-based, functional, and participation assessments for individuals with SMA in a standardized, safe and reliable manner.
4. Interprets and applies results of the impairment-based, functional, and participation assessments to the management of the individual with SMA.
5. Recommends appropriate assistive devices, seating and mobility equipment, and environmental modifications for the individual with SMA to achieve a positive maximal impact on function.
6. Provides patient/family-centered management.
7. Makes appropriate multi-disciplinary professional referrals.
8. Utilizes physiotherapy care guidelines for SMA patient management across the disease spectrum and lifespan.
9. Demonstrates safe handling skills during physiotherapy management of the individual with SMA across the disease spectrum and lifespan.

Do you have any general comments about the competencies in the patient management section?

**Communication Competencies**

1. Clearly and accurately receives and disseminates information in a respectful manner that considers situational needs and results in intended outcomes.
2. Effectively engages in interprofessional communication that positively affects patient outcomes.
3. Seamlessly and intuitively adapts to diverse verbal and non-verbal communication styles during anticipated and unanticipated patient and professional interactions.
4. Discriminates and incorporates appropriate strategies to engage in challenging encounters with patients and others to negotiate positive outcomes.

Do you have any general comments about the competencies in the communication section?

**Clinical Reasoning Competencies**

1. Synthesizes information gathered during the examination to form a movement diagnosis related to SMA.
2. Utilizes best practices in SMA regarding toward the interpretation of examination findings, the setting of goals and rehabilitation treatment priorities including patient/family input.
3. Incorporates evidence-based SMA practice in decision-making to determine and carry out treatment procedures and progression of intervention.
4. Actively envisions future scenarios with individuals with SMA based on an understanding of disease progression and impact of pharmaceutical and rehabilitation treatments.
5. Understands the ethical and practical dilemmas that impinge on both the conduct of pharmaceutical and

rehabilitation SMA treatments and their desired goals, and responds with a patient/family-centered focus.

Do you have any general comments about the competencies in the clinical reasoning section?

**Professionalism Competencies**

1. Identifies SMA resources and pursues areas of professional development that lead to continued competence.
2. Utilizes external feedback and self-reflection to improve SMA patient care.
3. Consistently demonstrates values of diversity, equity and inclusion in interactions with patients and families.
4. Develops solutions to ethical issues and understands their potential impact on patient outcomes, patient/therapist safety and public trust.
5. Promotes innovation in research and practice to advance the profession.
6. Serves as a SMA resource to provide feedback and expert guidance to the interprofessional community.

Do you have any general comments about the competencies in the professionalism section?

**Education Competencies**

1. Designs, directs, and implements educational activities for various members of the SMA community (including patients, families, other clinicians, and researchers)
2. Applies effective teaching strategies for cognitive, psychomotor, and affective domains of learning.
3. Adapts one's teaching style to reflect the learner: their level of experience, preferences, needs and goals.
4. Provides mentorship to advance the professional development of other SMA physiotherapists.

Do you have any general comments about the competencies in the education section?
