# Supplementary material for: Essential competencies for physical therapist managing individuals with spinal muscular atrophy: A delphi study
Source: PLoS One. 2021 Apr 22;16(4):e0249279. doi: 10.1371/journal.pone.0249279 (PMC8062020; doi:10.1371/journal.pone.0249279)
Supplement: S3 Appendix — (DOCX) [file pone.0249279.s003.docx]

Survey- Round Three

Thank you for your feedback and comments in the second round. All of the feedback was reviewed by the research team and the list was modified accordingly. There are now 35 competencies under 6 domains. In this third and FINAL round we ask that you rate whether or not you agree that a particular competency is essential for a physiotherapist/physical therapist working with an individual with SMA to demonstrate competency in. You will rate your agreement on a 5-point Likert scale ranging from Strongly Disagree to Strongly Agree. This round is relatively short and should take no longer than 15 minutes to complete.

We ask that you complete this survey by Monday, July 20th, 2020. We truly appreciate your time and expertise!

If you have questions at any time feel free to contact the primary investigators:

Jacqueline Montes, PT, EdD

Associate Professor of Rehabilitation and Regenerative Medicine in the Programs in Physical Therapy

Columbia University Irving Medical Center

617 West 168th Street, Room 347

New York, NY 10032

212-305-8916 phone

[Jm598@cumc.columbia.edu](mailto:Jm598@cumc.columbia.edu)

Jean Fitzpatrick Timmerberg, PT, PhD, MHS

Associate Director

Programs in Physical Therapy, Vagelos College of Physicians & Surgeons

617 West 168th Street, Georgian Building - 3rd Floor

New York, NY 10032

212-305-2814 phone

[jt2634@cumc.columbia.edu](mailto:jt2634@cumc.columbia.edu)

If you would like to download a PDF version of the domains and competencies, you can do so here:

Knowledge of Practice: Physiotherapists are experts in movement and function. They

demonstrate knowledge in the established and evolving evidence-based science related to

SMA. Physiotherapists integrate this unique knowledge and skills to provide quality care and

enhance the participation, health and wellbeing of their patients with SMA.

1. Demonstrates an understanding of typical development of the healthy individual and aging across the lifespan.

Strongly Disagree Disagree Neutral Agree Strongly Agree

1. Demonstrates an understanding of the pathophysiology of SMA. (i.e., SMN function within the cell, neuromuscular junction pathophysiologic abnormalities in SMA, mitochondrial function in SMA, spinal cord pathology in SMA, muscle pathology in SMA.

Strongly Disagree Disagree Neutral Agree Strongly Agree

1. Demonstrates an understanding of the SMA disease progression over time in each individual, and its impact on body functions and structure, including all body systems (e.g., musculoskeletal, respiratory, cardiovascular, gastrointestinal, neurological).

Strongly Disagree Disagree Neutral Agree Strongly Agree

1. Demonstrates an understanding of the multidisciplinary care guidelines for SMA and how they can be applied within one's (provider/patient's) current healthcare system.

Strongly Disagree Disagree Neutral Agree Strongly Agree

1. Demonstrates an understanding of physiotherapy care guidelines for SMA with regard to signs/symptoms, evaluations, and interventions.

Strongly Disagree Disagree Neutral Agree Strongly Agree

1. Demonstrates an understanding of the mechanism of action (pharmacodynamics) of pharmacologic treatments for SMA.

Strongly Disagree Disagree Neutral Agree Strongly Agree

1. Recognizes evolving phenotypes as newly-observed patterns of SMA disease progression diverge from classic SMA trajectories as a result of pharmacologic or other interventions.

Strongly Disagree Disagree Neutral Agree Strongly Agree

1. Do you have any comments about any of the competencies under the Knowledge of Practice domain?

Patient Management: As experts in movement and function, physiotherapists provide care for

individuals with SMA through the use of knowledge, skills and shared decision making with

patients and families and other professionals to optimize patients' outcomes.

1. Demonstrates active listening skills throughout all aspects of SMA patient management.

Strongly Disagree Disagree Neutral Agree Strongly Agree

1. Gathers a comprehensive medical and psychosocial history pertinent to SMA from the patient/family.

Strongly Disagree Disagree Neutral Agree Strongly Agree

1. Selects and administers appropriate, comprehensive impairment-based, functional, and participation assessments for individuals with SMA in a standardized, safe and reliable manner.

Strongly Disagree Disagree Neutral Agree Strongly Agree

1. Interprets and applies results of the impairment-based, functional, and participation assessments to the management of the individual with SMA.

Strongly Disagree Disagree Neutral Agree Strongly Agree

1. Recommends appropriate assistive devices, seating and mobility equipment, and environmental modifications for the individual with SMA to optimize function.

Strongly Disagree Disagree Neutral Agree Strongly Agree

1. Provides patient/family-centered management.

Strongly Disagree Disagree Neutral Agree Strongly Agree

1. Makes appropriate referrals to members of the multi-disciplinary team.

Strongly Disagree Disagree Neutral Agree Strongly Agree

1. Applies physiotherapy care guidelines for SMA patient management across the disease spectrum and lifespan.

Strongly Disagree Disagree Neutral Agree Strongly Agree

1. Demonstrates safe handling skills during physiotherapy management of the individual with SMA across the disease spectrum and lifespan.

Strongly Disagree Disagree Neutral Agree Strongly Agree

1. Do you have any comments about any of the competencies under the Patient Management domain?

Communication: As strong communicators, physiotherapists providing care for individuals

with SMA demonstrate interpersonal, verbal, nonverbal and written communication skills to

effectively exchange information and collaborate with patients, families, and other

professionals.

1. Clearly and accurately receives and disseminates information in a respectful manner that considers situational needs.

Strongly Disagree Disagree Neutral Agree Strongly Agree

1. Effectively engages in interprofessional communication that positively affects patient outcomes.

Strongly Disagree Disagree Neutral Agree Strongly Agree

1. Adapts to diverse verbal and non-verbal communication styles during anticipated and unanticipated patient and professional interactions.

Strongly Disagree Disagree Neutral Agree Strongly Agree

1. Selects and incorporates appropriate strategies to manage challenging encounters with patients and others.

Strongly Disagree Disagree Neutral Agree Strongly Agree

1. Do you have any comments about any of the competencies under the Communication domain?

Clinical Reasoning: Physiotherapists can critically translate evidence-based knowledge into

practice. They demonstrate the ability to organize, synthesize, integrate, and apply sound

clinical rationale for SMA patient management.

1. Synthesizes information gathered during the examination to form a movement diagnosis related to SMA.

Strongly Disagree Disagree Neutral Agree Strongly Agree

1. Utilizes current best practice guidelines for management of patients with SMA to interpret examination findings and to develop shared goals and treatment priorities with patient/family input.

Strongly Disagree Disagree Neutral Agree Strongly Agree

1. Incorporates SMA evidence-based practice to determine treatment procedures and progression of intervention.

Strongly Disagree Disagree Neutral Agree Strongly Agree

1. Anticipates future scenarios with individuals with SMA based on an understanding of disease progression and impact of pharmaceutical and rehabilitation treatments.

Strongly Disagree Disagree Neutral Agree Strongly Agree

1. Considers factors that may influence decision making with regards to pharmaceutical and rehabilitation treatments for SMA, and responds with a patient/family-centered focus.

Strongly Disagree Disagree Neutral Agree Strongly Agree

1. Do you have any comments about any of the competencies under the Clinical Reasoning domain?

Professionalism: Physiotherapists providing care for individuals with SMA, demonstrate a

commitment to life-long learning while working in the best interest of patients, colleagues,

society and the profession. They maintain high standards of behavior, exhibit appropriate

professional conduct, advocate for the patient, and adhere to ethical principles.

1. Identifies resources and pursues areas of professional development that lead to continued competence in SMA.

Strongly Disagree Disagree Neutral Agree Strongly Agree

1. Effectively and regularly utilizes external feedback and self-reflection to improve SMA patient care.

Strongly Disagree Disagree Neutral Agree Strongly Agree

1. Consistently demonstrates values of diversity, equity and inclusion in interactions with individuals with SMA and their families.

Strongly Disagree Disagree Neutral Agree Strongly Agree

1. Understands the potential impact of ethical issues in SMA on patient outcomes, patient/therapist safety and public trust and works to develop and implement solutions.

Strongly Disagree Disagree Neutral Agree Strongly Agree

1. Promotes innovation in SMA research and practice to advance the profession.

Strongly Disagree Disagree Neutral Agree Strongly Agree

1. Serves as a SMA resource to provide feedback and expert guidance to the interprofessional community.

Strongly Disagree Disagree Neutral Agree Strongly Agree

1. Do you have any comments about any of the competencies under the Professionalism domain?

Education: All physiotherapists are educators, teaching and mentoring various members of

the SMA community including patients, families, students, and other professionals.

1. Designs and directs educational activities for various members of the SMA community (including patients, families, other professionals.

Strongly Disagree Disagree Neutral Agree Strongly Agree

1. Implements effective teaching strategies for the various domains of learning (cognitive- knowledge, psychomotor hands on skills, and affective-moods, feelings, and attitudes).

Strongly Disagree Disagree Neutral Agree Strongly Agree

1. Adapts one's teaching style to reflect the learner: their level of experience, preferences, needs and goals.

Strongly Disagree Disagree Neutral Agree Strongly Agree

1. Provides mentorship to advance the professional development of other SMA physiotherapists.

Strongly Disagree Disagree Neutral Agree Strongly Agree

1. Do you have any comments about any of the competencies under the Education domain?
2. Do you have any additional comments or suggestions that you would like to add?
